# Draft Genome Sequences of Nine Campylobacter hyointestinalis subsp. lawsonii Strains

**DOI:** 10.1128/MRA.01016-18

**Published:** 2018-09-13

**Authors:** Xiaoming Bian, Steven Huynh, Mary H. Chapman, Christine M. Szymanski, Craig T. Parker, William G. Miller

**Affiliations:** a Department of Microbiology and Complex Carbohydrate Research Center, University of Georgia, Athens, Georgia, USA; b Produce Safety and Microbiology Research Unit, Agricultural Research Service, U.S. Department of Agriculture, Albany, California, USA; c Department of Biological Sciences, University of Alberta, Edmonton, Alberta, Canada; University of Maryland School of Medicine

## Abstract

With increasing reports of Campylobacter hyointestinalis species associated with human diseases, more genome sequences are required to understand the virulence mechanisms of this emerging pathogen. Here, we describe the genome sequences of nine C. hyointestinalis subsp. lawsonii strains.

## ANNOUNCEMENT

Campylobacter jejuni is one of the major causes of gastrointestinal illness worldwide (1–4), and other Campylobacter species are increasingly reported to be associated with diarrheal burden (5, 6). C. hyointestinalis is among these emerging pathogens and is commonly isolated from farm animals and associated with diarrhea, gastric adenocarcinomas, proliferative enteritis, and inflammatory bowel disease in humans through zoonotic infection (7–11). The complete genome sequences of two subspecies, C. hyointestinalis subsp. hyointestinalis and C. hyointestinalis subsp. lawsonii, were first published in 2016 (12), but additional C. hyointestinalis subsp. lawsonii genome sequences have not been published since then. Additional genome sequences are required for understanding the virulence mechanisms and developing molecular detection methods for this emerging pathogen. Here, we report the draft genome sequences of nine C. hyointestinalis subsp. lawsonii strains.

Nine C. hyointestinalis subsp. lawsonii strains, representing eight multilocus sequence typing (MLST) sequence types (STs) (13), including the type strain of the subspecies (CHY5 = LMG 14432 = NCTC 12901 = CCUG 34538), were selected for sequencing (Table 1). Single colonies from each strain were grown microaerobically for 48 h at 37°C on anaerobe basal agar supplemented with 5% laked horse blood. Genomic DNA was extracted from a loop (∼5 μl) of cells using the Wizard genomic DNA kit.

**TABLE 1:** Source information, accession numbers, and genome data for the nine C. hyointestinalis subsp. lawsonii strains

Strain	ST	Source	BioSample no.	BioProject no.	SRA no.	GenBank accession no.	Reads			Contigs			
							Total no.	Avg length (bases)	Coverage (×)	Total no.	No. >5,000 bp	% of genome in large contigs (Q40%)	Total sequence length (Mb)
CHY5^T^	8	Pig, stomach	SAMN09274393	PRJNA473765	SRP150729	QMAE00000000	1,572,688	238.5	209	86	57	97.7 (100)	1.786
RM9004	97	Pig, feces	SAMN09274394	PRJNA473766	SRP150725	QMAF00000000	1,060,682	237	143	68	38	97.9 (100)	1.747
RM9426	20	Pig, feces	SAMN09274395	PRJNA473767	SRP150727	QMAG00000000	1,179,661	237.2	153	76	47	97.7 (100)	1.823
RM9752	21	Pig, feces	SAMN09274396	PRJNA473771	SRP150726	QMAH00000000	1,809,534	238	241	71	43	97.6 (100)	1.785
RM9767	22	Pig, feces	SAMN09274397	PRJNA473772	SRP150728	QMAI00000000	1,184,803	237.3	155	82	49	97.3 (100)	1.808
RM10071	23	Pig, feces	SAMN09274398	PRJNA473773	SRP150732	QMAJ00000000	1,580,942	235.6	202	91	43	95.8 (100)	1.836
RM10074	22	Pig, feces	SAMN09274399	PRJNA473774	SRP150734	QMAK00000000	1,409,722	233.5	181	82	47	96.8 (100)	1.809
RM10075	24	Pig, feces	SAMN09274400	PRJNA473775	SRP150736	QMAL00000000	941,976	236.3	124	67	40	97.7 (100)	1.786
RM14416	37	Cow, feces	SAMN09274401	PRJNA473776	SRP150737	QMAM00000000	940,690	241.2	127	54	33	98.1 (100)	1.786

Genome sequencing was performed using the Illumina MiSeq platform (Table 1). For each genome, the 2 × 250-bp paired-end trimmed MiSeq reads (average length = 237 bp) were assembled using the Newbler assembler version 2.6. An average of 75 contigs were obtained for the nine C. hyointestinalis subsp. lawsonii genomes, with approximately 96 to 98% of each genome represented by large contigs of >5,000 bp. Nearly every base in these contigs had a quality score of ≥40. The coverage for each genome ranged from 124× to 241×. All sequencing reads were deposited in the NCBI Sequence Read Archive (SRA; Table 1).

The average total sequence length for the nine C. hyointestinalis subsp. lawsonii genomes was 1.796 Mb (Table 1), and the G+C contents for the nine genomes ranged from 33.3 to 33.5%. These data are consistent with the previously sequenced genome of C. hyointestinalis subsp. lawsonii strain LMG 15993 (1.753 Mb, 33.6% G+C content) (12). Each genome was annotated using the NCBI Prokaryotic Genome Annotation Pipeline (PGAP) and the RAST (Rapid Annotations using Subsystem Technology) version 2 server (http://rast.nmpdr.org) (14, 15). PGAP analysis identified an average of 1,814 putative protein-coding genes and 39 to 44 tRNAs per genome.

All strains possess genes encoding factors for bacterial defense, antimicrobial resistance, virulence, and DNA exchange, including homologs of colicin V bacteriocins, the Campylobacter multidrug efflux (Cme) pump, a macrolide efflux (MacAB) transporter, systems for heavy metal resistance, group II capsular polysaccharides, nonulosonic acid biosynthesis, N-linked protein glycosylation, DNA transfer mechanisms (Tra, Trb, T4SS), and the cytolethal distending toxin (CdtABC), while some strains produce homologs of zonula occludens toxin, prophage remnants, and tetracycline resistance proteins. All these features should be examined in greater detail to understand the pathogenesis of this emerging Campylobacter species.

### Data availability.

This whole-genome shotgun project has been deposited at DDBJ/ENA/GenBank under the accession numbers QMAE00000000 to QMAM00000000. The versions described in this paper are the first versions, QMAE01000000 to QMAM01000000.
